# Fulminant Adrenal Crisis as the Initial Healthcare System Presentation of Adolescent Cerebral X‐Linked Adrenoleukodystrophy

**DOI:** 10.1002/cns.70914

**Published:** 2026-04-27

**Authors:** Junqiu Zhou, Wei Meng, Wei Zhao, Chenguang Shen

**Affiliations:** ^1^ Department of Neurology Baiyun District People's Hospital of Guangzhou Guangzhou Guangdong China; ^2^ BSL‐3 Laboratory (Guangdong), Guangdong Provincial Key Laboratory of Tropical Disease Research School of Public Health, Department of Laboratory Medicine, Zhujiang Hospital, Key Laboratory of Infectious Diseases Research in South China Southern Medical University, Ministry of Education Guangzhou China

**Keywords:** adrenal crisis, case report, X‐ALD

## 1 Case Presentation

A 19‐year‐old previously healthy male was admitted to hospital with an 11‐h history of seizures and coma. Retrospective review of his history indicated that he had not received systematic medical evaluation previously. Long‐standing diffuse hyperpigmentation was present, and he may have experienced nonspecific symptoms such as fatigue and decreased appetite; however, these were overlooked by both the patient and his family. On admission, the patient was in a deep coma (GCS 5; E2V1M2), exhibiting decerebrate posturing and chemosis. Diffuse brown‐black hyperpigmentation was noted across his skin and oral mucosa. Vital signs included a temperature of 40.2°C, heart rate of 142 bpm, and blood pressure of 134/88 mmHg. Neurological examination revealed increased muscle tone in all four limbs and bilateral Babinski signs.

**FIGURE 1:**
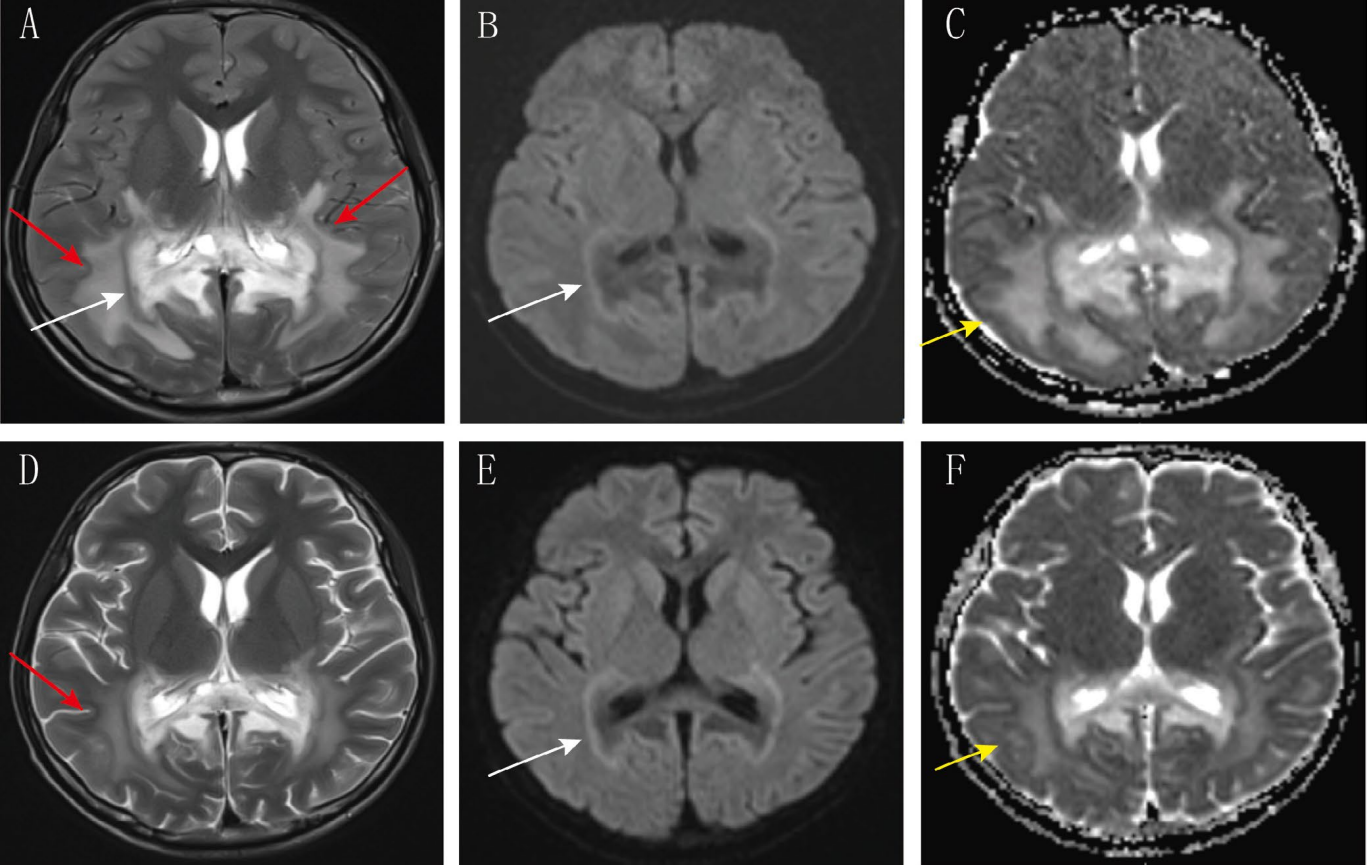
Dynamic changes in occipital lobe white matter lesions before and after treatment. Top row: T2‐weighted, DWI, and ADC images. (A–C) show symmetrical parieto‐occipital white matter involvement (red arrows) with splenial extension and corticospinal tract involvement. A central arcuate T2‐hypointense band (white arrow) is visible within the lesion, accompanied by high signal on DWI and low signal on ADC, suggesting an active inflammatory demyelinating core. Peripheral high signal on ADC (yellow arrow) represents reversible reactive edema due to increased blood–brain barrier permeability. Follow‐up images (D–F) show marked absorption of peripheral high signal intensity (yellow arrow) and reduced lesion volume (red arrow), while the central necrotic zone medial to the diffusion‐restricted area persists (white arrow). This indicates reversible vascular edema and partial resolution of the inflammatory demyelinating core, whereas the inner necrotic zone has evolved into irreversible structural damage.

**FIGURE 2:**
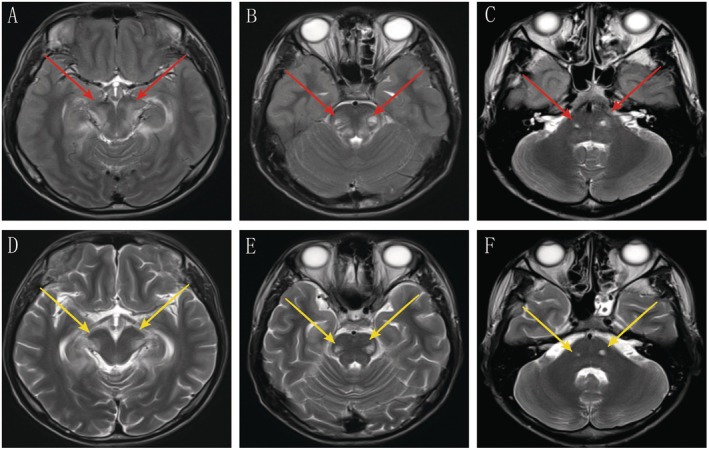
Bilateral Corticospinal Tract Lesions and Post‐Treatment Dynamic Changes. At admission (A–C), axial T2‐weighted images reveal symmetrical hyperintensity along the bilateral corticospinal tracts, accompanied by peripheral patchy vascular edema (red arrows) and blurred margins. Follow‐up imaging at 1 week (D–F) demonstrated marked resolution of the peripheral edema with sharpened fascicular borders (yellow arrows), suggesting reversible vascular permeability‐related edema. However, demyelinating lesions within the fascicles persisted, consistent with residual structural changes following resolution of the inflammatory response.

A brain MRI performed on the day of admission demonstrated characteristic symmetric white matter lesions in the parieto‐occipital regions, conforming to the pattern of symmetrical parieto‐occipital white matter involvement with splenial extension and corticospinal tract involvement as described in the 2023*X‐ALD MRI Reporting Consensus* (*Neurology* 2023;100:e1923) (Figures 1A–C and 2A–C). Lumbar puncture revealed an elevated opening pressure (240 mmH_2_O) and a markedly elevated CSF protein level (2887 mg/L). Laboratory findings included leukocytosis (12 × 10^9^/L), elevated CRP (168 mg/L), hyponatremia (134 mmol/L), and hypoglycemia (3.2 mmol/L). Management with meropenem, electrolyte correction, and nutritional support was initiated.

On hospital Day 3, endocrine results returned, showing a markedly elevated ACTH > 2000 pg/mL (reference: 7–64) and an inappropriately low cortisol level of 4.6 μg/dL (expected ≥ 18 μg/dL during stress). Combined with clinical symptoms, a diagnosis of primary adrenal insufficiency with acute decompensation (adrenal crisis) was confirmed. Therapy was immediately switched to intravenous hydrocortisone (100 mg twice daily), alongside intensified fluid resuscitation and electrolyte management. A repeat lumbar puncture showed reduced opening pressure (150 mmH_2_O) and decreased CSF protein (1415 mg/L), indicating restored blood–brain barrier integrity and reduced inflammatory exudation. ACTH levels decreased to 209 pg/mL, but cortisol rose only to 7.8 μg/dL, suggesting partial and likely irreversible adrenal dysfunction.

Follow‐up MRI 1 week later showed significant reduction of the peripheral hyperintensity and clearer demarcation of the lesions in the parieto‐occipital regions and corticospinal tracts (Figures 1D–F and 2D–F), indicating resolution of inflammation‐related vasogenic edema. However, the persistent abnormal signals in the core regions and within the tracts reflected irreversible demyelinating injury. This imaging evolution aligns with the characteristic radiological progression of X‐linked adrenoleukodystrophy (X‐ALD): the lesion originates in the parieto‐occipital white matter, demonstrating bilateral hemispheric involvement that spans the corpus callosum. It extends downward along the corticospinal tract, exhibiting a layered progression characterized by reversible peripheral oedema, persistent central inflammation, and deep fixed necrosis.

To quantify the extent of brain involvement and enable individual longitudinal comparison, we employed the MRI semi‐quantitative scoring system proposed by Loes et al. in 1994. Brain MRIs performed on admission and 1 week after treatment were scored. All images were independently assessed by two physicians with experience in neuroimaging (one neuroradiologist and one neurologist) who were blinded to the clinical follow‐up outcomes. In cases of scoring discrepancies, a consensus was reached through joint review of the images and discussion based on the original criteria to determine the final Loes score (Table 1).

**TABLE 1:** Longitudinal Loes MRI severity score in cerebral X‐ALD.

Scoring domain	Structures assessed	Maximum score	Admission Loes	2‐Week follow‐up Loes
Supratentorial White Matter	Parieto‐occipital white matter (periventricular/central/subcortical)	3	3	2
	Frontal white matter (periventricular/central/subcortical)	3	2	1
	Anterior temporal white matter (periventricular/central/subcortical)	3	0	0
Corpus callosum involvement	Genu, body, splenium	3	2	2
Visual pathways	Optic radiation, lateral geniculate body, Meyer's loop	3	3	2
Auditory pathways	Medial geniculate body, brachium of inferior colliculus, lateral lemniscus, pontine auditory area	4	3	2
Projection fibers	Internal capsule, brainstem corticospinal tract/frontopontine tract	2	2	1
Other structures	Basal ganglia, cerebellum, anterior thalamus	3	2	1
Focal atrophy (including corpus callosum)	Parieto‐occipital, frontal, anterior temporal, cerebellum, brainstem, genu and splenium of corpus callosum	7	0	0
Global cerebral atrophy	Bilateral frontal horn ratio + third ventricle width	3	0	0
Total score		0–34	17	11

*Note:* The Loes score is calculated according to the original MRI semi‐quantitative scoring method proposed by Loes in 1994, primarily used to quantify the extent of white matter involvement in X‐ALD. This scoring system is based on T2‐weighted and FLAIR sequences, assessing supratentorial white matter, corpus callosum, visual and auditory pathways, projection fibers, and other deep brain structures. Each anatomical structure is scored on an “all‐or‐none” basis: 0 = normal; 0.5 = unilateral or questionable involvement; 1 = bilateral definite involvement. MRI involvement includes T2 hyperintensity, T1 signal abnormality, or contrast enhancement; scattered, unspecified punctate hyperintense foci are not scored. Focal atrophy requires definite loss of brain parenchyma; global cerebral atrophy is quantified by the bifrontal horn ratio and third ventricle width (maximum 3 points). During longitudinal follow‐up, if a region shows definite imaging improvement, the score for that region is halved; if subsequent follow‐up shows continued improvement, it can be halved again. The total Loes score ranges from 0 to 34.

Subsequent genetic testing identified a hemizygous nonsense mutation in the ABCD1 gene, c.1073C>G (p.S358*), a known pathogenic variant, confirming the diagnosis of adolescent cerebral X‐linked adrenoleukodystrophy (ACALD). Family studies indicated the patient's sister was a heterozygous carrier, while his father did not carry the mutation. Maternal genetic data were unavailable.

The patient was discharged on a maintenance dose of prednisone (7.5 mg/day). At discharge, his speech was clear, but he required assistance for walking. Two months later, he discontinued glucocorticoid therapy without medical supervision. Thirteen months after discharge, he experienced a recurrent adrenal crisis triggered by an infection and died despite resuscitative efforts.

## 2 Discussion

X‐ALD is caused by mutations in the ABCD1 gene, leading to impaired peroxisomal beta‐oxidation of very long‐chain fatty acids (VLCFAs). The resulting lipotoxic injury can affect both the adrenal cortex and central nervous system white matter, clinically presenting as primary adrenal insufficiency (PAI) and inflammatory demyelination [1]. The cerebral phenotype is most common in childhood, while the ACALD is relatively rare [2, 3]. This case demonstrates that X‐ALD can present as a life‐threatening adrenal crisis during the patient's first medical encounter. This suggests that X‐ALD should be considered a priority differential diagnosis in young males with unexplained PAI or adrenal crisis.

The patient presented with a clear diagnosis of PAI upon admission and had progressed to a crisis. Adrenal crisis does not result from acute glandular necrosis but rather represents a metabolic decompensation triggered by a stressor (e.g., infection) causing a sudden surge in cortisol demand against a background of severely compromised hormonal reserve, leading to a “supply–demand” imbalance. In this case, the patient's consciousness rapidly improved (GCS increased from 5 to alert) following intravenous hydrocortisone after conventional anti‐infective and circulatory support proved ineffective. This suggests his coma was primarily due to reversible metabolic cerebral dysfunction rather than permanent structural injury [4, 5]. Pathophysiologically, cortisol deficiency can disrupt cerebral glucose metabolism, increase blood–brain barrier permeability, and cause electrolyte shifts, leading to an energy crisis and cerebral edema. Glucocorticoid replacement not only restores glucose utilization and suppresses NF‐κB‐mediated inflammation but also rapidly improves neuronal excitability and consciousness levels by stabilizing cerebral microvascular endothelium and mitochondrial function. This mechanism fully explains the reversible nature of the rapid consciousness recovery observed [6, 7, 8].

Neuroimaging further revealed the layered nature of the pathological injury. MRI showed bilateral parieto‐occipital white matter lesions. The lesion core exhibited DWI hyperintensity/ADC hypointensity, suggesting cytotoxic edema and active demyelination, while the peripheral T2/FLAIR hyperintensity with elevated ADC reflected vasogenic edema. Follow‐up imaging demonstrated significant resolution of the peripheral edema, while the core injury persisted, forming a “dichotomous response”: hormone replacement could reverse the metabolic disturbances and vasogenic edema secondary to the crisis, but had no significant effect on the established structural demyelination. The patient's initially elevated intracranial pressure and markedly high CSF protein indicated acute blood–brain barrier disruption and active inflammatory exudation. Post‐treatment, the intracranial pressure normalized and CSF protein decreased but remained elevated; these changes mirrored the MRI evolution where peripheral edema resolved but core lesions persisted. This reveals a “dichotomous response” pattern: partial restoration of blood–brain barrier function coexisting with ongoing structural demyelination.

Hormone replacement is the life‐saving cornerstone during the crisis but does not alter the natural history of the cerebral disease. Allogeneic hematopoietic stem cell transplantation (HSCT) is currently the only intervention proven to slow neurological progression, with efficacy highly dependent on early intervention. At the time of diagnosis, the patient was already in a deep coma with extensive cerebral lesions (Loes score 17). Following resolution of the adrenal crisis after treatment, follow‐up MRI showed a decrease in the Loes score to 11. However, upon evaluation after the condition had stabilized, the score remained above the optimal threshold for transplantation (≤ 9), and the patient had already developed definitive neurological deficits. Based on current comprehensive assessment criteria combining imaging and neurological function, the patient had missed the optimal window for transplantation [9, 10]. By the time of diagnosis, this patient was in deep coma with extensive cerebral involvement (Loes score > 17), clearly missing the optimal transplant window. This case highlights a critical dilemma in clinical management: a difficult‐to‐bridge time gap exists between rescuing the metabolic crisis and preserving brain structure. To address this challenge, establishing a multidisciplinary parallel assessment workflow is crucial—conducting neurological imaging and genetic evaluation simultaneously with crisis management could secure a critical intervention window for transplant‐eligible patients.

This study has limitations: The patient was critically ill upon admission, and plasma VLCFA analysis was not performed. Although ABCD1 gene sequencing confirmed the diagnosis, VLCFA analysis remains an important screening tool for X‐ALD, and the absence of this test reduces the completeness of the diagnostic workup [3]. This situation also reflects the practical challenges of early recognition of rare genetic diseases in the context of acute and critical illness.

This case demonstrates the rare presentation of ACALD with adrenal crisis as the initial manifestation, underscoring the importance of concurrently assessing neurological involvement during acute metabolic decompensation. Through enhanced mechanism understanding and early multidisciplinary intervention, clinicians can achieve a better balance between “metabolic rescue” and “structural preservation.” Such cases underscore the need for rapid coordination between crisis recognition, imaging evaluation, and genetic confirmation to improve patient survival rates and long‐term neurological outcomes.

## Author Contributions

All authors contributed to the conception and design of the study. Material preparation, data collection, and analysis were performed by J.Z., W.M., C.S., and W.Z. The initial draft was written by J.Z. and W.M., with all authors providing feedback on earlier versions of the manuscript. All authors reviewed and approved the final version.

## Funding

The authors have nothing to report. This work was supported by the Science and Technology Program of Guangzhou [No. 2025A04J6161].

## Consent

This study was conducted in accordance with the principles of the Declaration of Helsinki. The patient was in a comatose state upon admission and died during the follow‐up period. Written informed consent was obtained from the patient's sister, who served as the next of kin, confirming her consent for the patient's participation in this study and the publication of this case report. This study has been approved by the Institutional Review Board/Ethics Committee of Baiyun District People's Hospital, Guangzhou City, Guangdong Province, China (Approval Number: BYRY‐BLBG‐202501‐01).

## Conflicts of Interest

The authors declare no conflicts of interest.

## Data Availability

The data that support the findings of this study are available from the corresponding author upon reasonable request.
